# Genome-wide discovery of long intergenic noncoding RNAs and their epigenetic signatures in the rat

**DOI:** 10.1038/s41598-017-13844-9

**Published:** 2017-11-01

**Authors:** Aimin Li, Zhong-Yin Zhou, Xinhong Hei, Newton O. Otecko, Junying Zhang, Yajun Liu, Hongfang Zhou, Zhiqiang Zhao, Lei Wang

**Affiliations:** 1 School of Computer Science and Engineering, Xi’an University of Technology, Xi’an, Shaanxi 710048 China; 2 State Key Laboratory of Genetic Resources and Evolution, Kunming Institute of Zoology, Chinese Academy of Sciences, Kunming, Yunnan 650223 China; 3 Kunming College of Life Science, University of Chinese Academy of Sciences, Kunming, Yunnan 650204 China; 4 School of Computer Science and Technology, Xidian University, Xi’an, Shaanxi 710071 China; 5 Higher Technology College, Xi’an University of Technology, Xi’an, Shaanxi 710048 China

## Abstract

Long intergenic noncoding RNAs (lincRNAs) play a crucial role in many biological processes. The rat is an important model organism in biomedical research. Recent studies have detected rat lincRNA genes from several samples. However, identification of rat lincRNAs using large-scale RNA-seq datasets remains unreported. Herein, using more than 100 billion RNA-seq reads from 59 publications together with RefSeq and UniGene annotated RNAs, we report 39,154 lincRNA transcripts encoded by 19,162 lincRNA genes in the rat. We reveal sequence and expression similarities in lincRNAs of rat, mouse and human. DNA methylation level of lincRNAs is higher than that of protein-coding genes across the transcription start sites (TSSs). And, three lincRNA genes overlap with differential methylation regions (DMRs) which associate with spontaneously hypertensive disease. In addition, there are similar binding trends for three transcription factors (*HNF4A*, *CEBPA* and *FOXA1*) between lincRNA genes and protein-coding genes, indicating that they harbour similar transcription regulatory mechanisms. To date, this is the most comprehensive assessment of lincRNAs in the rat genome. We provide valuable data that will advance lincRNA research using rat as a model.

## Introduction

Long noncoding RNAs (lncRNAs) are a set of transcripts that are longer than 200 nt and do not encode proteins. LncRNAs accomplish a remarkable variety of biological functions such as epigenetic modification, transcriptional regulation and cell fate determination^1–3^. Recent studies associate lncRNAs with many diseases^4^. For example, *Mhrt* lncRNA offers protection against heart failure and hypertrophy^5^, while a lncRNA gene in the *HOX* loci, *HOTAIR*, is associated with cancer invasiveness^6^. Rat has extensively been utilised as a model for researching several devastating human diseases like Parkinson’s, Alzheimer’s, Peyronie’s, Huntington and degenerative joint disease^7–12^. Whereas 58,648 lncRNA genes have been retrieved from about 43 Tb of RNA-seq data of the human genome^13^, only a small proportion of rat lincRNA genes have been uncovered^14–16^. For instance, Amy Leung *et al*. annotated 466 lncRNA transcripts from two rat vascular smooth muscle cells^14^, Feng Wang *et al*. uncovered 2,761 lncRNA transcripts corresponding to 1,620 gene loci from six rat tissues^15^ and Kathirvel Gopalakrishnan *et al*. identified 3,272 lncRNA transcripts from three rat strains^16^. While these studies report the locations of the lncRNAs, their gene structures are unclear. Moreover, the enormous discrepancy in the number of lncRNAs characterised in the genomes of human and rat reveals that numerous rat lncRNA genes remain unidentified, considering that rats have similar genome size as human. Fortunately, rapid development of next-generation sequencing technologies and bioinformatics algorithms has generated a large amount of data that supports an accurate discovery of lncRNA transcripts in a large-scale manner.

Investigations of the role of lincRNAs in epigenetics have revealed that epigenetic mechanisms such as DNA methylation and histone modification in mammalian lincRNAs have differential patterns compared to protein-coding genes^17^. We have also previously demonstrated that methylation level of pig lincRNA genes is higher than that of protein-coding genes^18^. Overall, DNA methylation is a defining feature of mammalian cellular identity and is essential for diverse biological processes^19,20^. Therefore, profiling DNA methylation across the rat genome, as a biological model for biological studies, is vital for in-depth understanding of the effects of epigenetics^21^. A few studies have described epigenetic patterns in specific rat protein-coding genes^22,23^. However, at a global scale, particularly in and around transcription start sites (TSSs) of lncRNA genes, these epigenetic patterns have scarcely been studied^22,23^. Transcription factors (TFs) are central to transcriptional regulation of gene expression^24^. Recent development of chromatin immunoprecipitation followed by sequencing (ChIP-seq) technologies has enhanced the capability for genome-wide identification of TF-binding sites^25^. Several studies have shown that TF-binding signals around the TSSs of genes are predictive of gene expression levels^26,27^. Although the expression and transcriptional regulation of protein-coding have been well characterised, such aspects in lncRNAs are still in their infancy. In rats, the TF-binding patterns of lincRNAs remain unclear.

Here, we report a large-scale identification and characterisation of rat lincRNAs. From a comprehensive dataset of more than 100 billion rat RNA-seq reads from 64 independent studies, we identify a high-confidence set of 39,154 lincRNA transcripts in 19,162 loci. Our analyses show that rat lincRNAs have similar sequence and expression characteristics with other mammals. In addition, there are differential DNA methylation patterns between lincRNA genes and protein-coding genes in the rat genome. Interestingly, both lincRNAs and protein-coding genes have similar TF-binding patterns around TSSs. To facilitate future research on lincRNAs, we avail an open-access database named RatTransc. RatTransc hosts the lincRNA genes we identified, the expression profiles derived from 320 samples across eleven tissues (SRP037986)^28^ and 18 single-cell transcriptomes (SRP041119)^29^, as well as other useful functions. The RatTransc database is available at http://www.ibiomedical.net/rattransc/. The rat lncRNA landscape can serve as a useful resource for medical research using the rat as a model, and provide valuable biomarkers for disease diagnosis.

## Results

### Identification of lincRNA genes in the rat

**Figure 1:**
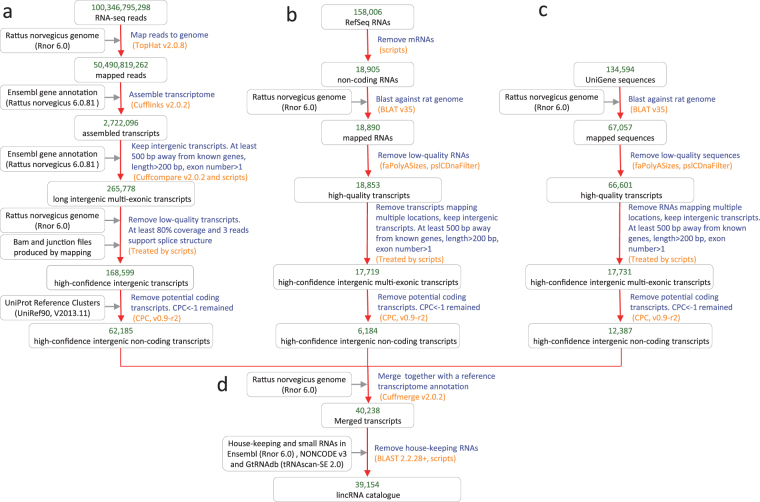
Flowchart describing the identification of lincRNAs. Processing of (**a**) RNA-seq datasets, (**b**) RefSeq datasets, (**c**) UniGene datasets, and (**d**) merging lincRNAs derived from RNA-seq, RefSeq and UniGene.

We collected 64 independent RNA-seq datasets. These included both single- and paired-end 12 to 151 bases long reads sequenced on Illumina platforms, totalling to about 100 billion reads from 1,289 samples and an average of about 78 million reads per sample (Supplementary Table S1). For a comprehensive retrieval of lincRNA genes in the rat genome, we developed a bioinformatics pipeline that integrates RNA-seq datasets with predetermined annotations from Ensembl, RefSeq and UniGene (see Methods, Fig. 1a,b,c and d). The pipeline involves five main steps: (1) aligning to reconstructed transcripts, (2) filtering low-quality transcripts, (3) keeping long intergenic multi-exonic transcripts, (4) evaluating coding potential of transcripts and (5) eliminating house-keeping RNAs. This approach is similar to the one we have applied in our previous studies on domestic animal lncRNAs^30,31^.

From the RNA-seq datasets, we derived 62,185 putative lincRNAs (Fig. 1a). Additionally, we identified 6,184 and 12,387 putative lincRNAs from RefSeq and UniGene annotations, respectively (Fig. 1b and c). After merging these transcripts using Cuffmerge and ruling out house-keeping genes, we obtained an assembly of 39,154 nonredundant lincRNA transcripts from 19,162 loci (Fig. 1d). The number is far more than Ensembl lincRNA annotation but has a high proportion of overlap with the Ensembl lincRNAs (Supplementary Figure S1). It also represents the most comprehensive lincRNA genes identification from the rat genome (Supplementary Dataset 1; http://www.ibiomedical.net/rattransc/).

### Sequence features of lincRNA genes

**Figure 2:**
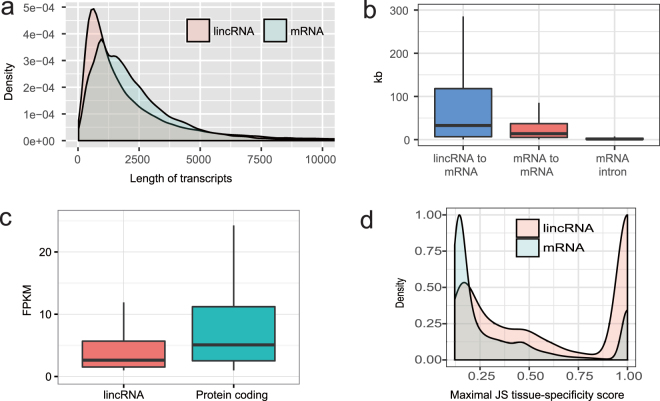
Structure and expression features of lincRNAs. (**a**) Size distribution of lincRNAs and protein-coding transcripts. In general, lincRNA transcripts are shorter than protein-coding. (**b**) Comparison of the mRNA-lincRNA intervals, mRNA-mRNA intervals, and sizes of mRNA introns. (**c**) LincRNAs have lower expression than protein-coding genes. FPKM, fragments per kilobase of exon per million fragments mapped. (**d**) Comparison of the distribution of tissue-specificity scores between lincRNAs and coding genes. Shown are distributions of maximal tissue-specificity scores (JS scores) calculated for each gene across the eleven tissues for coding genes (blue), lincRNAs (pink). More lincRNAs exhibit tissue-specific expression patterns (JS > 0.5).

To determine the basic sequence characteristics of newly identified rat lincRNA genes, we compared these novel lincRNA genes with protein-coding genes annotated by Ensembl (Rnor_6.0.81). In general, these lincRNA transcripts are short in comparison with protein-coding transcripts (Fig. 2a), which is in accordance with observations in other mammals such as human, mouse and pigs^13,30,32^. The distances between lincRNA genes and their neighbouring protein-coding genes are greater than the median distances between pairs of neighbouring coding gene (median of 32,678 nt for mRNA-lincRNA intervals compared to 13,630 nt for mRNA–mRNA intervals, Mann-Whitney, *P*-value < 2.2 × 10^−16^) (Fig. 2b). And, the distances between lincRNA genes and their protein-coding gene neighbours are larger than the intronic lengths of protein-coding genes (Mann-Whitney, *P*-value < 2.2 × 10^−16^). Thus, these lincRNAs are not likely to be undetected exons of protein-coding genes. The novel lincRNA genes we identified are independent transcripts, not unannotated exons of protein-coding genes.

### Expression characteristics of lincRNA genes

Yu and colleagues have reported a rat RNA-seq transcriptomic BodyMap covering 11 organs and four developmental stages (juvenile, adolescence, adult and aged) in both sexes of 320 Fischer rats (SRA accession number: SRP037986). However their study mainly focused on protein-coding genes^28^. Based on this transcriptomic dataset, we catalogued the expression profiles of 22,092 protein-coding genes annotated in Ensembl as well as the 19,162 novel lincRNA genes we annotated. The expression of these lincRNAs appears to be lower than those of protein-coding genes (Fig. 2a).

To assess the lincRNA gene expression profiles of the 320 rat samples, we carried out a hierarchical cluster analysis (Supplementary Figure S2). Clustering and heatmaps were generated in R Bioconductor using the heatmap.2 function of the gplots package (https://CRAN.R-project.org/package = gplots). According to unsupervised clustering of expression profiles, this analysis shows that more than half the lincRNA genes form tissue-specific expression patterns. Additionally, we performed clustering analysis of expression of protein-coding genes (Supplementary Figure S3), showing similar results to those of other mammals previously published^33^. Together, these results demonstrate that lincRNA genes are expressed in a more tissue-specific manner than protein-coding genes.

To further characterise the tissue-specific expression of lincRNA genes, we calculated each gene’s tissue-specificity score based on an entropy metric which relies on Jensen-Shannon (JS) divergence^34^ (Supplementary Note 1). The tissue-specificity metric (0-1) measures the similarity between a gene’s expression across all eleven organs and its expression in only one tissue. In an extreme case, a gene is expressed in only one tissue, giving a JS score of 1, which is a perfect tissue-specific expression. Analyses show that lincRNA genes tend to be tissue-specific (JS > 0.5) in comparison to protein-coding genes, which is in agreement with the clustering analysis (Fisher exact test, *P*-value < 2.2 × 10^−16^) (Fig. 2d). Specifically, 4,456 lincRNA genes exhibit tissue-specific expression across 11 tissues (adrenal gland, brain, heart, kidney, liver, lung, muscle, spleen, thymus, and testes or uterus). The distributions of tissue-specific lincRNAs in the 11 tissues are markedly different (Supplementary Figure S4). For instance, the largest category by total number of tissue-specific lincRNAs occurs in testes, which represents 48.9% of all tissue-specific lincRNAs we observed. This is consistent with published results from a survey of lincRNAs, in which testes was ranked the highest among 24 human tissues^34^. Interestingly, 762 of the total number of genes have high specificity for brain and 82 for heart tissues, respectively (Supplementary Table S2). This highlights the suitability of the rat as an excellent model for lincRNA-based research on nervous behaviour and hypertension.

### GO analysis of protein-coding genes neighbouring tissue-specific lincRNAs

Previous reports indicate that lncRNA genes possibly interact with chromatin proteins to positively or negatively regulate the expression of neighbouring genes^34–36^. To investigate the potential biological functions of rat tissue-specific lincRNA genes, we performed GO enrichment for the 4,456 lincRNA genes - which exhibit tissue-specific expression across the 11 tissues in the study of SRP037986. For each tissue, we first extracted the nearest neighbouring Ensembl-annotated protein-coding genes to the lincRNAs. We then used Ensembl Biomart (http://www.ensembl.org/biomart/martview) to get human homologues of these protein-coding genes and checked the enrichment of their Gene Ontology (GO)^37^ functional terms using DAVID Bioinformatics Tool^38^. In the rat brain, the GO category of neurological process exhibits significant enrichment. Additionally, protein-coding genes in close proximity to lincRNA genes with brain specific expression are associated with synapse function and behaviour. These findings emphasise why the rat is an excellent model for neuroscience research.

### Patterns of DNA methylation of rat lincRNAs

**Figure 3:**
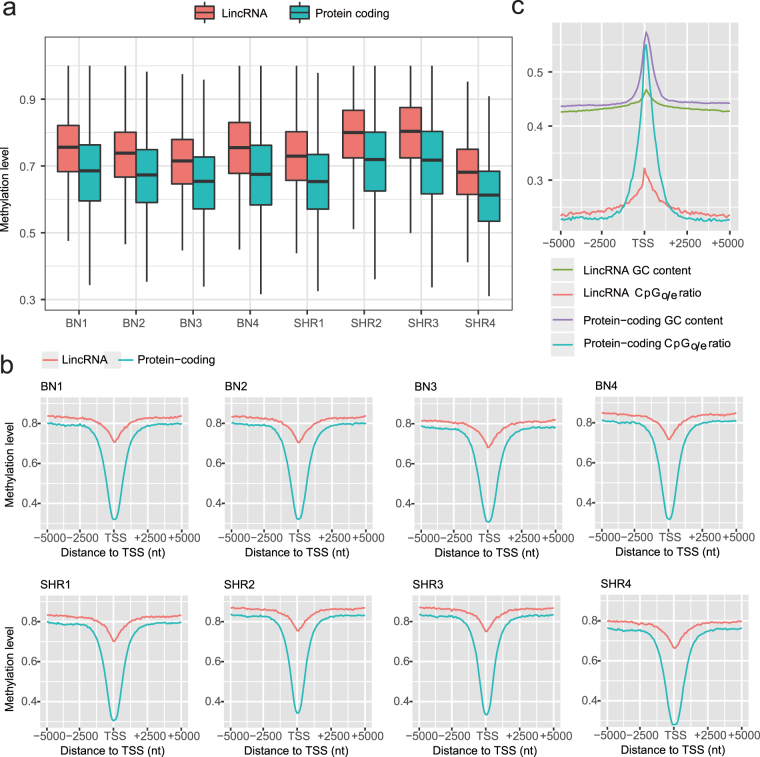
Characteristics of DNA methylation. (**a**) Protein-coding genes have lower methylation levels than lincRNAs. This figure shows the methylation levels inside protein-coding and lincRNA genes in four spontaneously hypertensive rats (SHR1, SHR2, SHR3 and SHR4), models of cardiovascular disease, and four Brown Norway (BN) control rats (BN1, BN2, BN3 and BN4). (**b**) DNA methylation patterns around TSSs of lincRNA and protein-coding genes. Both lincRNAs and protein-coding genes exhibit similar V-shaped curves while protein-coding genes present lower methylation around TSSs. Distributions of methylation levels were calculated in 100-bp bins, 5-kb upstream and downstream from the TSSs. (**c**) Distribution of GC content and CpGo/e ratio around TSSs of lincRNA and protein-coding genes. Distributions were calculated in 100-bp sliding windows, 5-kb upstream and downstream from the TSSs.

Michelle Johnson *et al*. have published whole-genome bisulfite sequencing (BS-seq) of the Brown Norway (BN) control strain and the spontaneously hypertensive rats (SHR) to test for association between methylation and pathophysiological phenotypes^22^. Our analysis of the BS-Seq dataset of BN and SHR strains revealed lower methylation levels in protein-coding genes than in lincRNAs (Kolmogorov-Smirnov, *P*-value < 2.2 × 10^−16^) (Fig. 3a). This corresponds with the fact that expression level of lincRNA genes is generally lower than that of protein-coding genes.

To further analyse the methylation differences between lincRNAs and mRNAs, we investigated the methylation patterns near TSSs using the BS-seq data. Methylation is lowest around TSSs of lincRNAs and protein-coding genes (Fig. 3b). Previous studies on human and pigs using MeDIP-seq data have reported that methylation levels of lincRNAs are higher than those of protein-coding genes in the regions of TSSs^17,39^. They further state that methylation patterns of protein-coding genes present V-shaped curves, whereas lincRNAs’ exhibit growing tendencies around TSSs. In this study, we found that both lincRNAs and protein-coding genes display similar V-shaped curves around TSSs in eight rats (Fig. 3b). Methylation levels of lincRNAs are higher than those of protein-coding genes at TSSs regions (Fig. 3b). In TSS regions, methylation of lincRNAs show different tendencies among rats, human and pigs.

To explore the reason why lincRNA and protein-coding genes have different methylation patterns, we compared GC content and CpG observed/expected ratio of lincRNAs and protein-coding genes at TSSs. This analysis shows that lincRNA and protein-coding genes have similar tendencies of GC content at TSSs (Fig. 3c). However, protein-coding genes display a higher observed/expected ratio as compared with lincRNAs, corresponding to their methylation levels. This observation differs from what we found in pigs^39^, suggesting that rats and pigs have diverse regulatory mechanism. Overall, this finding can clarify why most lincRNAs have lower expression levels than protein-coding genes.

### LincRNAs associated with cardiovascular disease

To detect lincRNA genes that have biological functions in cardiovascular disease, we re-analysed the BS-seq data published by Michelle Johnson *et al*.^22^ using the DSS package^40^. A total of 1,182 differentially methylated regions (DMRs) are identified, and three DMRs overlap with lincRNA genes (*RatTranscG007202* vs. dmr1178, *RatTranscG007905* vs. dmr1161, *RatTranscG015025* vs. dmr1142) (Supplementary Table S3). The three lincRNA genes might be associated with cardiovascular disease.

### Binding intensity profile of transcription factors at TSSs

**Figure 4:**
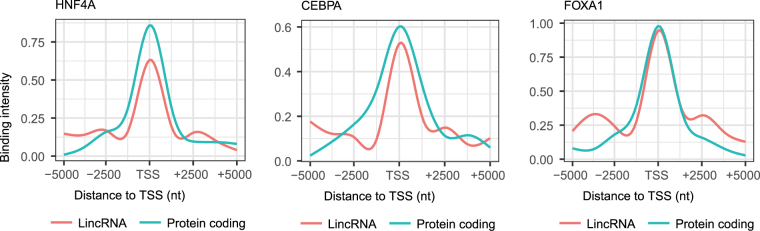
Transcription factor binding patterns. Transcription factor binding intensities are similar between lincRNAs and protein-coding genes around the TSSs. The *x*-axis represents the relative distance to the TSS, and the *y*-axis represents the mean binding intensities of TFs (*HNF4A*, *CEBPA* and *FOXA1*).

Transcription factors are central to the regulation of gene expression. We compared the binding intensity profiles of TFs to lincRNA and protein-coding TSSs using published ChIP-seq data of three tissue-specific TFs (*HNF4A*, *CEBPA* and *FOXA1*) from rat liver tissues^41^. Our analyses show that lincRNAs and protein-coding genes have nearly the same TF-binding profiles at TSSs (Fig. 4). *HNF4A*, *CEBPA* and *FOXA1* all exhibit upside-down V-shaped patterns at the TSSs. These results indicate that lincRNAs and protein-coding genes use similar regulatory mechanism, suggesting that lincRNAs have essential role in development.

## Discussion

The rat is an important animal model for biological studies, especially in neurobiological and cardiovascular disease research. LincRNA genes play important roles in many biological processes. To comprehensively identify lincRNA genes, we collected 64 RNA-seq datasets, RefSeq annotations and UniGene data, from which we identified 39,154 lincRNA transcripts in 19,162 intergenic regions. Currently, this is the most comprehensive analysis of lincRNAs in the rat genome. In human, 58,648 lincRNAs have been identified from RNA-seq datasets retrieved from 25 studies^13^. The integrity of lincRNA transcripts assembled from RNA-seq data largely depends on the depth of sequencing and the expression levels of lincRNA transcripts in the related samples^13^. The depth of sequencing and the expression levels of lincRNA transcripts vary across the samples we used in this study. Similar to our previous study^26,27^, we employed strict criteria including the coverage of junctions and exons to filter transcripts. Therefore, the lincRNA dataset identified in this research contains high-quality transcripts and presents a valuable resource for functional investigations utilising rat as a biological model.

The expression pattern of rat lincRAN genes shows similarity to those of other mammals. Interestingly, many lincRNA genes in the rat display tissue-specific expression. Particularly, testes lead in the number of tissues-specific expression of lincRNA genes, which is consistent with a previous study^42^, and indicates that the rat is an excellent model for reproduction biology and health research. Additionally, a previous study proposed that rat represent a model for behavioural studies^43,44^. In our study, many brain tissue-specific linRNAs proximal to the protein-coding genes associate with neurological processes. Thus, this research will enrich knowledge on the candidate lincRNA genes applicable for neuroscience investigations.

Previous studies indicate that lincRNA genes have different epigenetic patterns from those of protein-coding genes^20,21^. Consistent with our previous study in pigs^21^, we have observed higher methylation levels around TSS of lincRNA genes compared to protein-coding genes. Contrary to the methylation levels of pig lincRNAs which show growing trends in TSS regions, the methylation trends of the rat lincRNAs in TSS regions display an upside-down V-shaped trend. However, the rat lincRNAs in TSS regions have a similar trend to the protein-coding genes. The differential DNA methylation trends at TSSs between rats and pigs may result from different DNA methylation sequencing methods. BS-seq has higher resolutions than the MeDIP-seq used in the pig study^21^. Thus, the DNA methylation trends across TSS regions in rats could be more accurate than those of pigs. We found similar TF-binding patterns between lincRNA genes and protein-coding genes for three TFs, *HNF4A*, *CEBPA* and *FOXA1*. This is an indication of similar transcriptional regulation for lincRNA genes and protein-coding genes. This is also the first comparison of the TFs binding sites on a genome-wide scale between rat lincRNA genes and protein-coding genes.

In summary, we report the most comprehensive lincRNA genes in various tissues using hundreds of billions of RNA-seq reads of the rat genome. We show that rat lincRNAs have similar characteristics with other mammals, and a number of lincRNA genes show preferential tissue expression. In addition, we characterised the epigenetic patterns between lincRNA genes and protein-coding genes, showing their differences in DNA methylation patterns and similarity in transcriptional factor binding patterns. We also report the overlapping of rat lincRNA genes with three DMRs linked with cardiovascular disease. This study provides important insights and data resources for future large-scale functional experiments using rat as an animal model.

## Methods

### Datasets used for identification of rat lincRNAs

To derive a more comprehensive set of lincRNAs that includes previous annotations, we used Cuffmerge to integrate the RNA-seq-derived lincRNAs with the predetermined set of non-coding RNAs annotated by NCBI RefSeq and UniGene.

We have collected all rat RNA-seq datasets produced by Illumina platforms. The datasets are listed in Supplementary Table S4 and all of them can be downloaded from NCBI SRA database (https://www.ncbi.nlm.nih.gov/sra/). During our step-wise identification of lincRNAs, some datasets were eliminated since no lincRNAs could be extracted from them using our stringent criteria. Finally, 64 RNA-seq datasets containing 100,346,795,298 reads from 1,298 samples were retained for further analysis. Details of the 64 RNA-seq datasets are shown in Supplementary Table S1.

The NCBI UniGene and RefSeq.^45^ sequences have been proven to be extremely valuable for the identification of lincRNAs in plants and mammals^46,47^. RefSeq (Release 73) curates 18,905 rat noncoding RNAs. These data resources can be leveraged to expand the rat lincRNA catalogue. To minimise transcriptional noise, high quality and non-redundant sequences (UniGene) were used in our study. We collected rat unigenes from the NCBI UniGene database (Rattus norvegicus: UniGene Build #195)^48^.

### Analyses of the RNA-seq datasets

The high volume and complexity of RNA-seq datasets call for efficient, robust and statistically principled tools^49^. TopHat and Cufflinks have together gained prominence in RNA-seq studies for identifying new genes, splice variants, and comparing expression of genes and transcripts under different conditions^49^. Here, we aligned all the RNA-seq reads to the Rattus norvegicus 6.0 genome using TopHat v2.0.8^49^. The transcriptome of each sample was assembled from mapped reads using Cufflinks v2.0.2 with a reference transcriptome annotation – Ensembl Rattus norvegicus annotation 6.0.81^49,50^ (Fig. 1a). Cuffcompare v2.0.2 program in Cufflinks suite was used to map the newly assembled transcripts back to annotations of Rattus norvegicus 6.0.81. Intergenic transcripts, annotated with class code ‘*u*’ by Cuffcompare, were merged using Cuffmerge for each study. Read coverage is related to the quality of assembled transcripts^46,51^. To obtain high-confidence transcripts, we used two criteria to filter the RNA-seq-derived transcripts: RNA-seq read coverage on exons must be at least 80% for each transcript, and there must be at least three RNA-seq reads mapping to the predicted splice structure^30^.

### Analyses of the RefSeq and UniGene datasets

For the RefSeq and UniGene data, each sequence was first mapped to the Rattus norvegicus 6.0 genome using BLAT (the BLAST-like alignment tool) (v35)^52^. BLAT is more accurate and about 500 times quicker than other commonly used mRNA/DNA alignment tools, making it suitable for large-scale genomic projects. Low-quality contigs were then removed using the pslCDnaFilter program (http://hgdownload.cse.ucsc.edu/admin/exe/) with the parameters: ‘*-minId* = *0.95*, *-minCover* = *0.25*, *-globalNearBest* = *0.0025*, *-minQSize* = *20*, *-minNonRepSize* = *16*, *-ignoreNs-bestOverlap*’. Contigs that equally mapped to multiple locations of the genomes were removed from further analyses (Fig. 1b and c).

### Construction of final lincRNA catalogue

To derive a unique comprehensive set of lincRNAs, we used the Cuffmerge v2.0.2 utility within the Cufflinks package to integrate the RNA-seq, RefSeq and UniGene derived putative lincRNAs (Fig. 1d).

To rule out house-keeping RNAs (including tRNAs, snRNAs and snoRNAs), putative lincRNAs were aligned to house-keeping RNA databases using BLASTN with the parameters: ‘*-evalue 1e-10 -perc_identity 80*’^53^. The house-keeping lncRNA databases include the tRNA database downloaded from the Genomic tRNA Database (http://gtrnadb.ucsc.edu/)^54^; the tRNA, snRNA and snoRNA database from the Ensembl Database^50^; and the snRNAs and snoRNAs collected from NONCODE v3.0^55^. LincRNA candidates that have significant (*P*-value < 1.0^−10^) alignment with house-keeping RNAs were not included in further analyses.

We also used the following criteria to identify lincRNAs: (1) The retained transcripts have at least two exons, in other words, they are multiple-exonic transcripts, (2) the lengths of transcripts are more than 200 nucleotides, (3) the transcripts which are located at least 500 bp away from neighbouring protein-coding or house-keeping genes are classified as intergenic transcripts, (4) the coding potential of intergenic transcripts was assessed using the Coding Potential Calculator (CPC)^56^, and only those transcripts with a CPC score of less than −1 were defined as noncoding genes.

### Expression profile of lincRNA genes

Reads from the study with the SRA accession number of SRP037986^28^ were mapped to the rat genome (Rnor 6.0) by TopHat v2.0.8 using the parameter ‘*–no-novel-juncs’*. Mapped reads were used to quantify the expression of the protein-coding and lincRNA genes using two independent software packages. SummarizeOverlaps v1.10.0 was used to calculate counts of mapped reads for each gene with the default mode of ‘Union’^57^. Cufflinks v2.0.2 was employed to obtain FPKM expression values with the parameter ‘*-G’*. Read mapping and expression quantification were run independently for each sample. We ran these three programs on the gene models of our GTF annotation and did not attempt to identify new transcripts in this step. The lincRNA gene expression measures are available at our RatTransc database.

### Analyses of DNA methylation patterns of rat lincRNAs

A BS-seq dataset, which included four Norway rat samples (BN) and four spontaneously hypertensive rats (SHR), was downloaded from the NCBI GEO database (accession number is ERP002215)^22^. Raw sequencing reads were filtered using Trimmomatic v0.33 with default parameters and then aligned to the Rattus norvegicus 6.0 genome sequences using Bismark v0.14.3 with the parameter ‘*–X 1000*’^58^. Bismark is a robust tool that conducts both read mapping and methylation calling in BS-seq data in a quick flexible step^58^. Therefore we extracted the methylation level of each cytosine from the aligned reads using the Bismark methylation extractor under standard parameters. The methylation level (%) for any given genomic interval refers to the ratio of the number of BS-Seq ‘methylated’ bases aligned to any genomic cytosine in that interval to the number of methylated or unmethylated bases aligned to the same.

To compare the methylation level of lincRNA genes to that of protein-coding genes in each sample, we first counted the number of methylated and unmethylated bases in each gene. We then used the equation $M=m/(m+u)$, where *m* is the number of methylated bases and *u* unmethylated, to calculate methylation level of each gene. *M* is zero if *m* = 0.

To explore the methylation patterns around TSSs, we took the regions of 5 kb downstream to 5 kb upstream of TSSs into account. Each 10 kb region was divided into 100 bins with equal lengths. For each bin, we used the following equation to calculate methylation levels.

1 $${M}_{ti}={m}_{ti}/({m}_{ti}+{u}_{ti}),\,t=1,\,{\rm{...}},\,n,\,i=1,\,{\rm{...}},\,100.$$

where *n* is the number of lincRNA or protein-coding transcripts, *M*_*ti*_ is the methylation level of the *i*-th bin of the transcript *t*, *m*_*ti*_ is the number of methylated bases in the *i*-th bin of the transcript *t*, and *u*_*ti*_ is the unmethylated. The average methylation level of each bin among lincRNA or protein-coding transcripts is defined as:

2 $${V}_{i}=\sum _{t=1}^{n}{M}_{ti}/n,\,i=1,\,{\rm{...}},\,100.$$

### Identification of DMRs from whole genome BS-seq reads

We identified differentially methylated regions (DMRs) across the entire genome between BN and SHR rats. Specifically, we first transformed the format of output of the Bismark methylation extractor and fed them into the Bioconductor package DSS (Dispersion Shrinkage for Sequencing data)^40,59^. We then performed statistical test for differential methylation loci (DML) without smoothing by calling DMLtest function in the Bsseq bioconductor package. Finally, with the test results, we called DMRs using the callDMR function with the parameter ‘*p* = *0.01*’.

### Analyses of binding sites of transcription factors

In order to analyse the binding profile of the three TFs to lincRNA and protein-coding TSSs, we extracted binding signals at each nucleotide position 5 kb up and downstream of TSSs and compared the signal tendencies among them. In particular, the ChIP-seq dataset was obtained from NCBI SRA database (accession number: ERP002078)^41^. Raw sequencing reads were mapped against the Rattus norvegicus 6.0 genome assembly using bowtie2^60^ with default parameters. The binding signals of each nucleotide position were extracted from aligned reads using SAMtools depth (https://github.com/samtools/samtools) under standard parameters.

We then considered the regions 5 kb up and downstream of TSSs. Each region was segmented into 100 sub-regions with the size of 100 bp. For each sub-region, we used the following equation to calculate binding intensity:

3 $${I}_{ti}=\sum _{p=1}^{100}{C}_{tip}/100,\,t=1,\,{\rm{...}},\,n,\,i=1,\,{\rm{...}},\,100$$

Where *n* is the number of lincRNA or protein-coding transcripts, *I*_*ti*_ is the average binding intensity of the *i*-th sub-region of the transcript *t*, and *C*_*tip*_ is the binding signals of the *p*-th nucleotide position in the *i*-th sub-region of the transcript *t*. The average binding intensity of each sub-region among lincRNA or protein-coding transcripts is defined as:

4 $${B}_{i}=\sum _{t=1}^{n}{I}_{ti}/n,\,i=1,\,{\rm{...}},\,100.$$

### Data availability

The datasets generated during the current study are available in the RatTransc repository, http://www.ibiomedical.net/rattransc/.

## Electronic supplementary material

Supplementary Material

Supplementary Table S1

Supplementary Table S2

Supplementary Table S3

Supplementary Table S4

Supplementary Dataset
