# Supplementary material for: Genome-wide discovery of long intergenic noncoding RNAs and their epigenetic signatures in the rat
Source: Sci Rep. 2017 Nov 1;7:14817. doi: 10.1038/s41598-017-13844-9 (PMC5665958; doi:10.1038/s41598-017-13844-9)
Supplement: Supplementary file 1 — Supplementary Material [file 41598_2017_13844_MOESM1_ESM.pdf]

Genome-wide discovery of long intergenic noncoding RNAs and their epigenetic signatures in the rat

Aimin Li, Zhong-Yin Zhou, Xinhong Hei, Newton O. Otecko, Junying Zhang, Yajun Liu, Hongfang Zhou, Zhiqiang Zhao & Lei Wang

Calculation of tissue-specificity scores (JS scores)

We used the approach developed by Moran Cabili *et al.*[1] to identify tissue-specific lincRNA and protein-coding genes. This approach is an entropy-based assessment that evaluates the consistency between a gene's expression profile and another predefined profile that represent an extreme case in which a gene is expressed in only one of 11 tissues. This specificity measurement is based on the Jensen-Shannon divergence (JSD)[2]. The Jensen–Shannon divergence is a method of measuring the similarity between two probability distributions. The JSD of two discrete probability distributions, p^1, p^2, is defined to as:

$$JSD(p^1, p^2) = H(\frac{p^1 + p^2}{2}) - \frac{H(p^1) + H(p^2)}{2}, \tag{1}$$

where H is the Shannon entropy of a discrete probability distribution:

$$p = (p_1, p_2, ..., p_{11}),\ 0 \leq p_i \leq 1,\ i = 1, 2, ..., 11,\ and \sum_{i=1}^{11} p_i = 1,$$

$$H(p) = -\sum_{i=1}^{11} p_i \log(p_i) \tag{2}$$

The square root of the Jensen–Shannon divergence is a metric often referred to as Jensen-Shannon distance[2]. The distance between two tissue expression profiles, e^1 and e^2, $e^i = (e_1^i, e_2^i, ..., e_{11}^i)$, is defined to be

$$JS_{distance}(e^1, e^2) = \sqrt{JSD(e^1, e^2)}. \tag{3}$$

The tissue specificity of a gene's expression profile, e, across 11 tissues with respect to tissue t can then be defined as

$$JS_{specifity}(e \mid t) = 1 - JS_{distance}(e, e^t), \tag{4}$$

where e^t is a predefined expression profile that represents the extreme case in which a gene is expressed in only one tissue. Formally,

$$e^t = (e_1^t, e_2^t, ..., e_{11}^t),\ s.t.\ e_i^t = \begin{Bmatrix} 1 & if\ i = t \\ 0 & otherwise \end{Bmatrix}.$$

Finally, the tissue specificity score (JS score) of a gene is defined as the maximal tissue specificity score across all 11 tissues of the genes expression profile e:

$$JS_{specifity}(e) = \arg\max_t JS_{specifity}(e \mid t),\ t = 1, \ldots, 11 \quad (5)$$

Normalisation of expression vectors for tissue specificity calculation

To calculate the tissue specificity scores of a gene, we needed to convert the gene's expression vector to an abundance density (as the JS metric is applied on discrete probability distributions). To this end, we added a pseudo-count of 1 to the raw FPKM (fragments per kilobase of exons per million fragments mapped) expression vector of each gene and applied a log 2 normalization to obtain a non negative expression vector. We then normalised this expression vector to a density vector by dividing by the total expression counts. Formally:

$$V' = \frac{\log_2(V + 1)}{\sum_{i=1}^{11} \log_2(\upsilon_i + 1)},$$

where $V = (\upsilon_1, \upsilon_2, \ldots, \upsilon_{11})$ is the original raw FPKM abundance estimation of the gene and V' is the new normalised density vector.

References

1. Cabili, M.N. et al. Integrative annotation of human large intergenic noncoding RNAs reveals global properties and specific subclasses. *Genes & development* **25**, 1915-1927 (2011).
2. Fuglede, B. & Topsoe, F. Jensen-Shannon divergence and Hilbert space embedding. in *Information Theory, 2004. ISIT 2004. Proceedings. International Symposium on* 31 (IEEE, 2004).

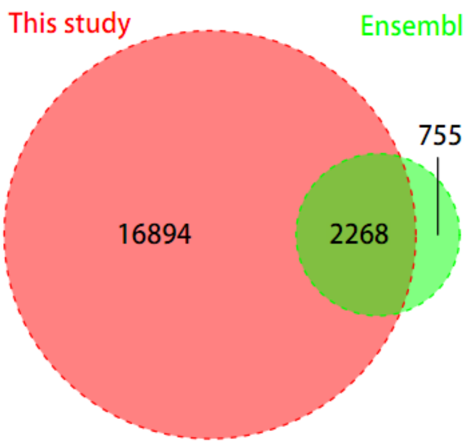

Supplementary Figure S1. Comparison of lincRNA numbers between this study (RatTransc) and Ensembl release 81.

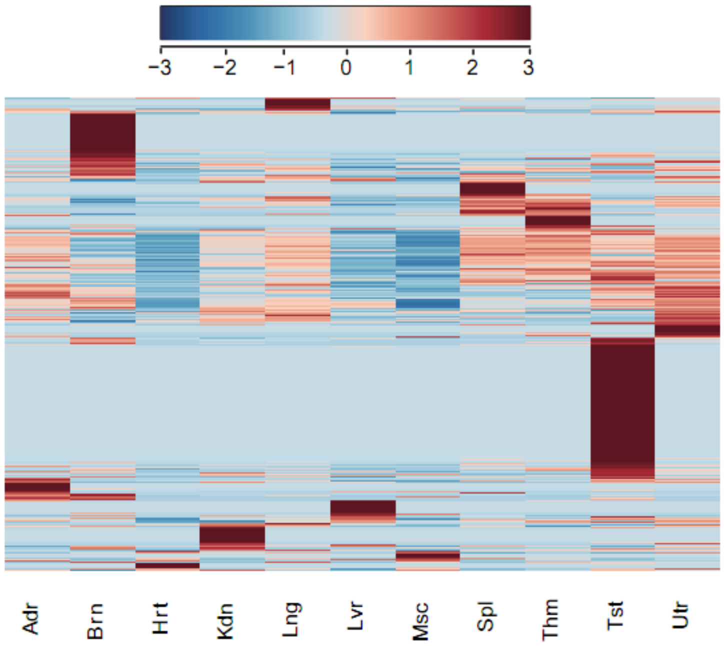

Supplementary Figure S2. Hierarchical clustering of the expression of lincRNA genes. Expression levels of lincRNA genes were derived from RNA-seq data based on 11 tissues and 320 samples (SRP037986). Adr: adrenal gland, Brn: brain, Hrt: heart, Kdn: kidney, Lvr: liver, Lng: lung, Msc: muscle, Spl: spleen, Thm: thymus, Tst: testes and Utr: uterus.

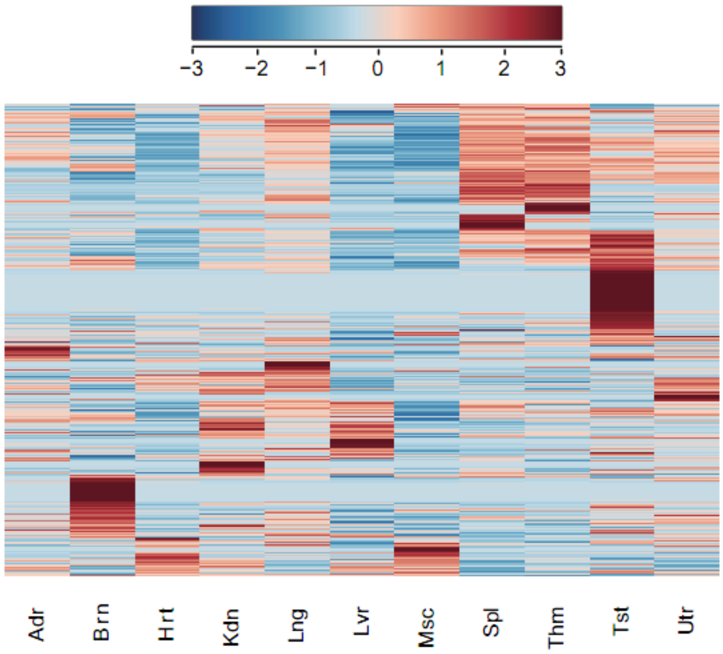

Supplementary Figure S3. Hierarchical clustering of the expression of protein-coding genes. Expression levels of protein-coding genes were derived from RNA-seq data based on 11 tissues and 320 samples (SRP037986). Adr: adrenal gland, Brn: brain, Hrt: heart, Kdn: kidney, Lvr: liver, Lng: lung, Msc: muscle, Spl: spleen, Thm: thymus, Tst: testes and Utr: uterus.

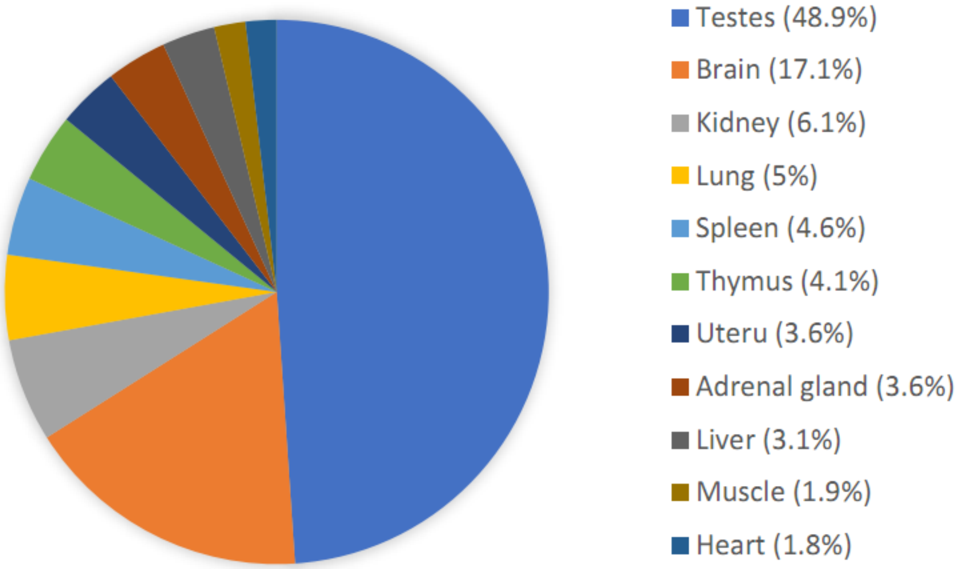

Supplementary Figure S4. Distribution of tissue-specific lincRNAs across 11 tissues.
